# Early plasma angiopoietin-2 is prognostic for ARDS and mortality among critically ill patients with sepsis

**DOI:** 10.1186/s13054-023-04525-3

**Published:** 2023-06-13

**Authors:** Carrie M. Rosenberger, Katherine D. Wick, Hanjing Zhuo, Nelson Wu, Yue Chen, Sharookh B. Kapadia, Alessander Guimaraes, Diana Chang, David F. Choy, Hubert Chen, Melicent Peck, Kathryn M. Sullivan, Serena Ke, Alejandra Jauregui, Aleksandra Leligdowicz, Pratik Sinha, Antonio D. Gomez, Kirsten N. Kangelaris, Kevin Delucchi, Kathleen D. Liu, Carolyn S. Calfee, Michael A. Matthay, Carolyn M. Hendrickson

**Affiliations:** 1 Human Pathophysiology and OMNI Reverse Translation, Genentech, Inc., 1 DNA Way, South San Francisco, CA 94080 USA; 2 Division of Pulmonary and Critical Care Medicine, University of California San Francisco, 505 Parnassus Ave, San Francisco, CA USA; 3 Infectious Diseases, Genentech, Inc., 1 DNA Way, South San Francisco, CA USA; 4 Human Genetics, Genentech, Inc., 1 DNA Way, South San Francisco, CA USA; 5 Early Clinical Development, Genentech, Inc., 1 DNA Way, South San Francisco, CA USA; 6 Zuckerberg San Francisco General, Division of Pulmonary and Critical Care Medicine, University of California San Francisco, 1001 Potrero Ave, San Francisco, CA 94110 USA; 7 Present Address: Krystal Biotech, Pittsburgh, PA USA; 8 Present Address: Department of Internal Medicine, University of California, Davis, Davis, CA USA; 9 Present Address: Department of Medicine, Division of Critical Care, Western University, London, ON Canada

## Abstract

Angiopoietin-2 (Ang-2) is associated with vascular endothelial injury and permeability in the acute respiratory distress syndrome (ARDS) and sepsis. Elevated circulating Ang-2 levels may identify critically ill patients with distinct pathobiology amenable to targeted therapy. We hypothesized that plasma Ang-2 measured shortly after hospitalization among patients with sepsis would be associated with the development of ARDS and poor clinical outcomes. To test this hypothesis, we measured plasma Ang-2 in a cohort of 757 patients with sepsis, including 267 with ARDS, enrolled in the emergency department or early in their ICU course before the COVID-19 pandemic. Multivariable models were used to test the association of Ang-2 with the development of ARDS and 30-day morality. We found that early plasma Ang-2 in sepsis was associated with higher baseline severity of illness, the development of ARDS, and mortality risk. The association between Ang-2 and mortality was strongest among patients with ARDS and sepsis as compared to those with sepsis alone (OR 1.81 vs. 1.52 per log Ang-2 increase). These findings might inform models testing patient risk prediction and strengthen the evidence for Ang-2 as an appealing biomarker for patient selection for novel therapeutic agents to target vascular injury in sepsis and ARDS.

## Introduction

Biomarker-guided patient selection has the potential to match specific biologic therapies with the most relevant pathophysiology and therefore with the greatest potential for benefit in acute respiratory distress syndrome (ARDS). The angiopoietin (Ang)-Tie pathway is a key regulator of vascular endothelial permeability that contributes to non-cardiogenic pulmonary edema in critically ill patients [1]. In ARDS and sepsis, antagonism of Tie2 signaling by soluble Ang-2 inhibitors disrupts endothelial cell tight junctions, leading to increased vascular permeability and inflammation (reviewed in [2]). Previous small prospective cohort studies of critically ill patients suggest an association between plasma Ang-2 and development of ARDS [3] and poor clinical outcomes in patients with sepsis, including those with respiratory failure [4, 5]. We hypothesized that higher plasma Ang-2 measured shortly after hospitalization (usually within 24 h) would be associated with worse clinical outcomes in a large cohort of critically ill patients with sepsis. More specifically, we hypothesized that early plasma Ang-2 would be associated with a higher incidence of developing ARDS and increased mortality after adjusting for relevant clinical and demographic features.

## Methods

We conducted a retrospective analysis of 758 prospectively enrolled septic patients admitted to the ICU from the emergency department in the EARLI (Early Assessment of Renal and Lung Injury study) cohort between October 2008 and August 2018 [3], 267 of whom developed ARDS according to the Berlin Definition within the first five days of ICU admission [6]. Sepsis was defined as meeting at least 2 SIRS criteria (> 38.0 °C or hypothermia < 36.0 °C, tachycardia > 90 beats/minute, tachypnea > 20 breaths/minute, leukocytosis > 12 × 10^9^ cells/L or leukopenia < 4 × 10^9^ cells/L) and confirmed positive microbiological culture or clinically diagnosed sepsis adjudicated by a research physician. The study was approved by the Institutional Review Board of the University of California, San Francisco. Procedures for informed consent have been previously described [3].

Plasma Ang-2 levels were measured using a Luminex™ multiplexed bead-based assay or ELISA (R&D™). Baseline (first available) Ang-2 values were used in all analyses, using log_e_-transformed values in analyses using Ang-2 as a continuous predictor variable. To assess the temporal relationship between elevated Ang-2 and ARDS development, we performed a subgroup analysis comparing Ang-2 levels between at-risk subjects who developed ARDS more than 24 h after plasma sampling (*n* = 78) to at-risk subjects who did not develop ARDS during our observation period (*n* = 408). This analysis excluded patients with ARDS identified within 24 h of ICU admission, for whom the timing of ARDS diagnosis was not clear, or for whom ARDS plasma was collected more than 24 h after enrollment.

Continuous variables are presented as median (IQR) and compared using Wilcoxon rank-sum tests. Categorical variables were compared using the Chi-square test. Adjusted logistic regression models (age and sex) were used to estimate the association between Ang-2 and 30-day mortality among all subjects. A separate analysis including an interaction term between log-transformed Ang-2 and ARDS was used to test whether ARDS mediated the association between Ang-2 and mortality. Adjustment for severity of illness scores was not included in these models as Ang-2 is on the same causal pathways as organ failures in our theoretical causal model. A two-sided P value less than 0.05 was considered statistically significant. Statistical analysis was performed with SAS 9.4 (SAS Institute Inc., Cary, NC, USA) and R.

## Results

Demographic characteristics of all septic patients and of those with sepsis and ARDS are presented in Table 1**.** At day 30, 25% of patients with sepsis in the overall cohort had died as compared with 39% of patients with both sepsis and ARDS. Baseline plasma Ang-2 was significantly associated with 30-day mortality in adjusted logistic regression models. In models adjusting for age and sex, a one unit change in log plasma Ang-2 was associated with 1.52-fold increase in the odds of death (95% CI 1.28–1.80) in the full cohort (of septic patients (*n* = 757) and a 1.81-fold increase (95% CI 1.37–2.40) in the patients with both sepsis and ARDS (*n* = 267) (Table 2). In a separate logistic regression model of 30-day mortality including an interaction term between Ang-2 and ARDS, there was not a statistically significant interaction (*p* = 0.071).

**Table 1:** Clinical and demographic data for the full cohort of septic patients and the nested cohorts of patients with sepsis and Berlin ARDS

Characteristic	Sepsis cohort (*n* = 757)	Berlin ARDS Cohort (*n* = 267)
Age, years (mean ± SD)	65 ± 16	66 ± 16
Male (*n*, %)	421, 56%	155, 58%
Female (*n*, %)	336, 44%	112, 42%
Caucasian (*n*, %)	374, 49%	132, 49%
African-American (*n*, %)	99, 13%	39, 15%
Asian (*n*, %)	191, 25%	64, 24%
Pacific Islander (*n*, %)	9, 1%	2, 1%
Native American (*n*, %)	5, 1%	1, 0%
Other (*n*, %)	79, 11%	29, 11%
Hispanic Ethnicity (*n*, %)	95, 13%	29, 11%
*Sepsis (n, %)*		
Pulmonary	361, 48%	169, 64%
Non-pulmonary	303, 40%	58, 22%
Both	62, 8%	26, 10%
Unclear	23, 3%	12, 5%
*Lung injury risk factor (n, %)*		
Direct lung injury risk factor	450, 59%	210, 79%
Indirect lung injury risk factor	307, 41%	57, 21%
None	0 (0%)	0 (0%)
Vasopressors on study day 1 (*n*, %)	435, 58%	186, 70%
APACHE III Score (mean ± SD)	98 ± 39	118 ± 39
PaO_2_/FiO_2_ (median, IQR) on day 1	173 (103, 273)	136 (71, 206)
Ventilator free days by 28 days	25 (3, 28)	18 (0, 25)

Hospital mortality: Death at hospital discharge. 60d mortality is inclusive of 30d mortality

Patient survival is defined as surviving past day 60 after study enrollment. Patients who died after day 60 are still included for calculating length of stay

**Table 2:** Higher Ang-2 is associated with mortality and ARDS in septic patients

	Odds ratio (95%CI)	
Sepsis cohort (*n* = 757)		
Death^1^	1.52 (1.28, 1.80)	< 0.001
ARDS^2^	1.32 (1.13, 1.53)	< 0.001
ARDS + Sepsis (*n* = 267)		
Death^3^	1.81 (1.37, 2.40)	< 0.001

^1^Death: 30-day hospital mortality. Final model was determined using a priori predictors for ARDS-related mortality. The final adjusted model includes: log(Ang-2) (shown above), age (odds ratio: 1.03; 95% CI 1.02–1.04; *p* < 0.001), and male sex (odds ratio: 1.06; 95% CI 0.78–1.45; *p* = 0.72)

^2^ARDS: ARDS by Berlin Definition within first 5 days. Final model was determined using a priori predictors for ARDS-related mortality. The final adjusted model includes: log(Ang-2) (shown above), age (odds ratio: 1.01; 95% CI 1.00–1.01; *p* = 0.27), and male sex (odds ratio: 1.26; 95% CI 0.94–1.69; *p* = 0.12)

^3^Same methodologies as full cohort. The final model includes: log(Ang-2) (shown above), age (odds ratio:), male sex (odds ratio: 1.27; 95% CI 0.74–2.17; *p* = 0.39)

Plasma samples were collected within 24 h of ICU admission for greater than 96% of patients, and within 48 h of for all patients for whom the timing of sample collection was recorded. Median (IQR) Ang-2 levels were higher among 78 sepsis patients in whom plasma was sampled more than 24 h prior to meeting Berlin ARDS diagnostic criteria compared to a group of 408 at-risk sepsis patients who did not develop ARDS during the first 5 days of hospitalization [7577 pg/ml (4216–16,699 pg/ml) vs*.* 6032 pg/ml (3009–11,821 pg/ml), *p* = 0.02].

Among all patients with sepsis, median Ang-2 levels were higher in patients requiring vasopressors on ICU Day 1 compared with patients who did not. Patients who required vasopressors on day 1 in ICU had Ang-2 median levels of 8426 (3763–16,000) compared to 5000 (2587–9334) for those patients who did not require vasopressors (*p* < 0.0001). In addition, patients in the highest quartile of Ang-2 compared to the lower three quartiles required a greater number of vasopressors (*p* < 0.0001), suggesting that baseline plasma Ang-2 may enrich for vasoplegia and shock.

## Discussion

To our knowledge, these findings represent the largest biomarker study of early measurements of plasma Ang-2 in a cohort of critically ill patients with physician-adjudicated ARDS and sepsis. Prior reports have found an association between Ang-2 and both organ failure and mortality in patients with respiratory failure [5] and an association between Ang-2 and the development of acute lung injury in smaller cohorts [3]. We present a novel finding with time-ordering data in critically ill patients with sepsis, demonstrating that higher plasma Ang-2 levels are associated with increased risk of developing ARDS, thereby strengthening the evidence for the importance of Ang-2 in the pathobiology of ARDS among critically ill patients with sepsis. The robust association between Ang-2 and 30-day mortality in patients with early sepsis, which is enhanced among a subgroup of patients with early sepsis who also have ARDS, suggests a conserved pathobiology role among heterogeneous critically ill patients. Our data support the potential role for measuring circulating Ang-2 to guide the delivery of therapeutic targets that target endothelial injury and may improve clinical outcomes in critical ill patients with sepsis, particularly those who are at risk of developing ARDS.

## Data Availability

The datasets used and/or analyzed during the current study are available from the corresponding author on reasonable request.
